# Depiction of dentatorubrothalamic tract fibers in patients with Parkinson’s disease and multiple sclerosis in deep brain stimulation

**DOI:** 10.1186/s13104-016-2162-8

**Published:** 2016-07-18

**Authors:** Ardian Hana, Anisa Hana, Georges Dooms, Hans Boecher-Schwarz, Frank Hertel

**Affiliations:** National Service of Neurosurgery, Centre Hospitalier de Luxembourg, Rue Barblé 25, 1210 Luxembourg, Luxembourg; Internal Medicine Rotterdam, Erasmus University Rotterdam, Rotterdam, The Netherlands; Service of Neuroradiology, Centre Hospitalier de Luxembourg, Luxembourg, Luxembourg

**Keywords:** Deep brain stimulation, White matter, Neurosurgery, White matter tract, Neurodegenerative disease

## Abstract

**Background:**

We wanted to depict fibers of the dentatorubrothalamic tract in patients with Parkinson’s disease and multiple sclerosis in order to use this knowledge for clinical routine and to show its relation to the corticospinal tract for deep brain stimulation. Fibers of these white matter tracts were depicted between February 2014 and February 2015 in nine patients of all ages. There were seven men and two women. The mean age was 60 years. We used a 3DT1 sequence for the navigation. Additional scanning time was less than 9 min. Both tracts were portrayed in all patients.

**Results:**

We were able to successfully portray these white matter tracts in all patients. We visualized the medial and lateral parts of the corticospinal tract by using a region of interest which covered the whole motor cortex. Furthermore we segmented the motor cortex. The fibers ran from this area of the brain through the internal capsule and they could be followed until their entry in the brainstem. The dentatorubrothalamic tract was smaller than the corticospinal tract. It was situated medio-posteriorly of the corticospinal tract. After decussation to the contralateral red nucleus it was localised next to the midline when it entered the motor cortex. From the thalamus on, it proceeds medially and posteriorly of the corticospinal tract further to the motor cortex. Depiction of the whole tract is essential for the differentiation of the dentatorubrothalamic tract with the corticospinal tract.

**Conclusions:**

The depiction of the dentatorubrothalamic tract might be useful for neurosurgeons when deep brain stimulation is planned. Knowing its relation to other white matter tracts can help physicians like neurosurgeons or neurologists avoid side effects and deal with patients with DBS. The position of the electrode might be crucial for a satisfactory outcome.

## Background

PD is a neurodegenerative disease associated with neuropsychiatric illness [1]. It is characterized by the death of dopaminergic neurons in the substantia nigra in pars compacta [1–3]. Patients present in particular with tremor, rigidity and postural instability [4, 5]. Furthermore patients may suffer from nonmotor disorders like depression, apathy, psychotic symptoms or sleep disorders [4, 6]. These symptoms might dramatically affect the quality of life not only of the patients but also of their families. Deep brain stimulation (DBS) is a surgical procedure which is used in the management of PD in patients with inadequate control of symptoms or with significant side effects from Levodopa [7, 8]. It delivers electrical pulses which are variable in amplitude, pulse width, and frequency, through permanently implanted electrodes [9, 10]. The importance of electrical current for signal transduction was already known to Galvani in the eighteenth century [7]. It took, however, more than two centuries until we were able to use it in the form of DBS for patients with PD. By means of DBS we are able to inhibit or activate a target in the brain [7]. Possible therapeutic targets include the subthalamic nucleus, the globus pallidus internus or the ventral intermediate nucleus of thalamus [7]. Most targets aim the improvement of motor symptoms of PD and the DBS is used for tremor of various origins [11] but there is still work necessary to be done for the correction of nonmotor symptoms of PD [7, 12]. Apart from that there still seems to be no concensus between neurosurgeons about the best target in cases of tremor dominant PD with some of them advocating the ventral intermediate nucleus of the thalamus and others preferring the subthalamic nucleus.

Diffusion tensor imaging (DTI) is a technique that enables neurosurgeons to portray white matter tracts (WMT) in vivo in healthy and non-healthy patients [13]. It can be used in a variety of diseases among others tumours, multiple sclerosis or neurodegenerative diseases like PD [14]. Furthermore DTI can reveal previously unexplained side effects of DBS towards distinct fiber tracts [11, 15]. Some of the WMT portrayed by the DTI include the visual pathway, corticospinal tract (CST) or the dentatorubrothalamic tract (DRT). The latter is the main fiber bundle that forms the superior cerebellar peduncle [11, 16]. Affection of this WMT might be the reason for tremor control in patients with PD in the posterior subthalamic region [11, 17]. The aim of our study was to visualize fibers of the DRT before surgery in patients with PD for everyday routine. Furthermore we deem necessary to know the relation between this WMT and the CST. The DRT seems to be a target point for patients who will undergo a DBS. Depending on the position of the electrode in the DRT, we might cause unwanted side effects due to the proximity of this WMT to the CST. Therefore, we portrayed both tracts in all our patients in order to have a better understanding of these two WMT.

## Methods

We included in our study between February 2014 and February 2015 nine consecutive patients. These were seven men and two women. There was no limit of age. The mean age was 60 years. There were eight patients suffering from PD and one patient suffering from MS. The MRI was performed in order to prepare an eventual DBS. An MRI-scan was acquired on a 3T General Electric SignaHDxt Scanner preoperatively. We performed a 3DT1-sequence to navigate. Furthermore T2-and DTI-sequences were carried out. The FOV was 200 × 200 mm^2^, slice thickness 2 mm, and the acquisition matrix of 96 × 96 yielding nearly isotropic voxels of 2 × 2 × 2 mm. 3T-MRI was performed strictly axial using 32 gradient directions and one b0-image. *b* value was 800 s/mm^2^. We used EchoPlanar-Imaging (EPI) and ASSET parallel imaging with an acceleration factor of 2. The additional scanning time was less than 9 min. One day after surgery a computed tomography of the brain was performed. We processed the DTI data on a standard commercial workstation (StealthViz, Medtronic Inc., USA). This software uses a straightforward fiber tracking approach known as fiber assignment by continuous tracking (FACT). Parameters for the tractographies were a maximum angle of 45° for the CST and 60° for the DRT, an FA Start Value of 0.10 and an ADC Stop Value of 0.20. First we segmented the motor cortex (MC) on both cerebral hemispheres. Then we tracked the CST from this area. A region of interest (ROI) was put in the brain stem. The fibers were tracked also from the brain stem to the MC. It is possible to use a ROI in place of the segmented area of the MC. For the DRT we acted in a similar way. The fibers were tracked from the dentate nucleus (DN) of the cerebellum to the red nucleus of the contralateral hemisphere and later to the MC. Therefore, we used two ROI. One of them was put in the DN of the cerebellum and the other one in the contralateral red nucleus. As mentioned before it is possible to use another ROI instead of the segmented area of the MC. In that case this white matter tract would be portrayed using three ROI. The ROI measured approximately 1 cm in the red nucleus and DN of the cerebellum whereas the ROI for the MC measured approximately the size of the MC.

## Results

**Fig. 1:**
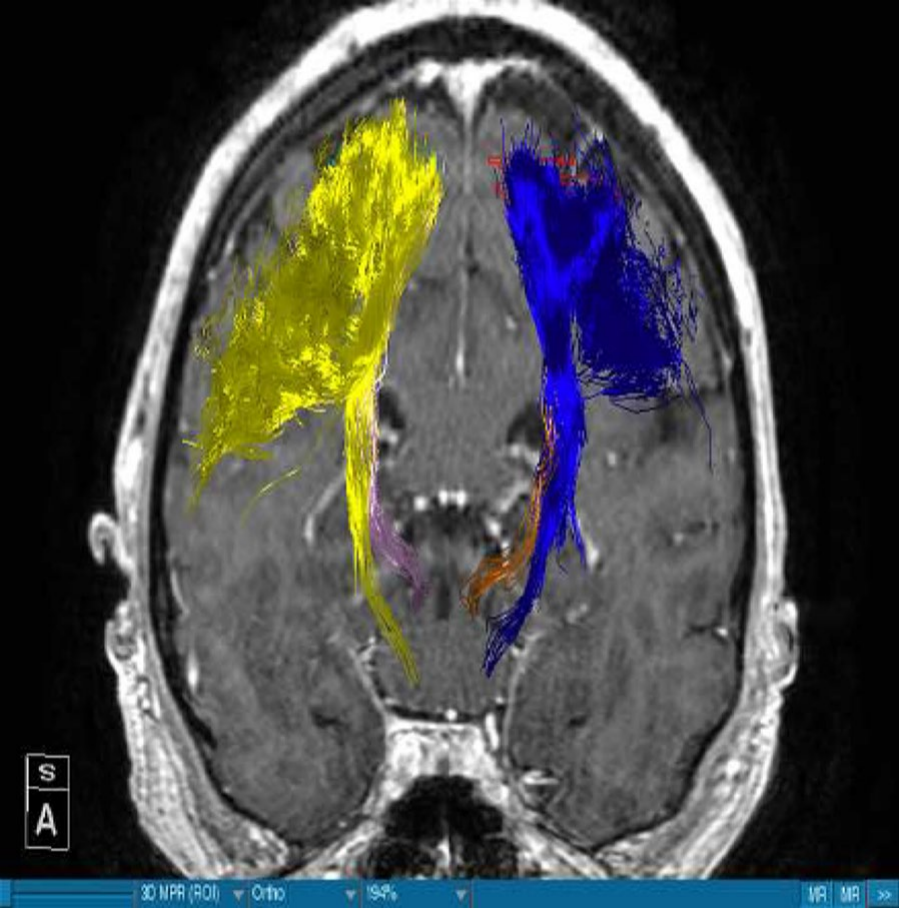
T1-image, anterior view, Parkinson’s disease. *Blue* left corticospinal tract, *Yellow* right corticospinal tract, *Gold* left dentatorubrothalamic tract, *Bright purple* right dentatorubrothalamic tract

**Fig. 2:**
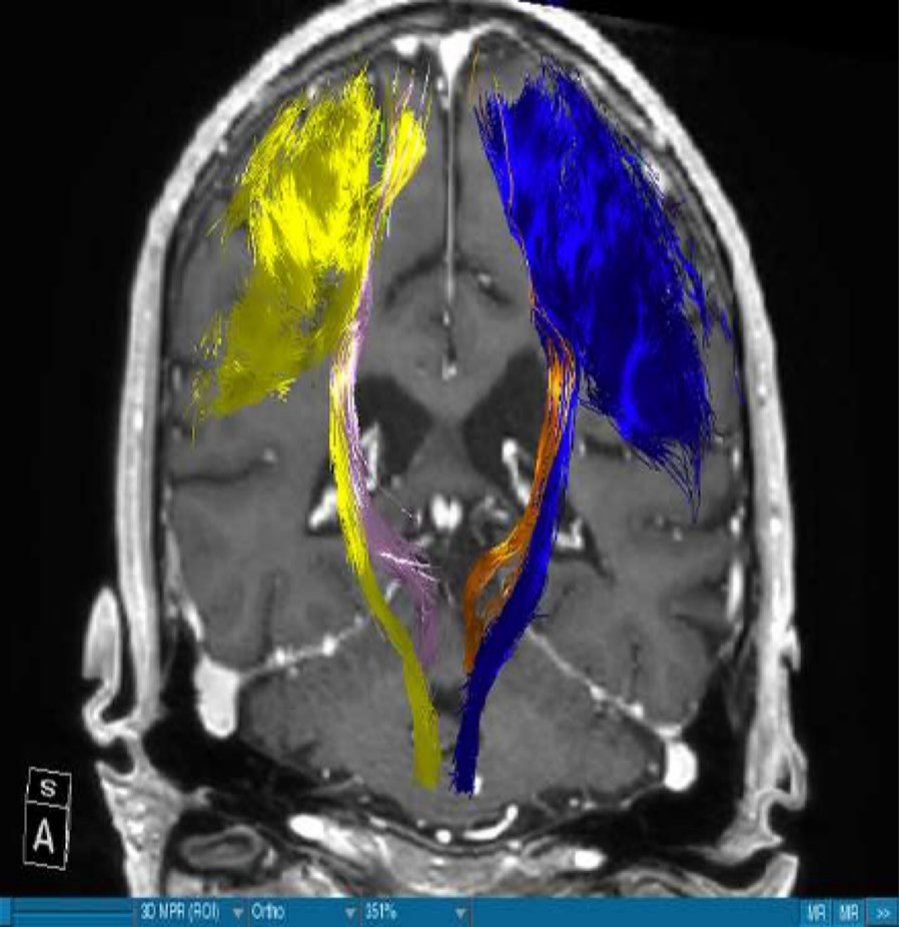
T1-image, anterior view, multiple sclerosis. *Blue* left corticospinal tract, *Yellow* right corticospinal tract, *Gold* left dentatorubrothalamic tract, *Bright purple* right dentatorubrothalamic tract

**Fig. 3:**
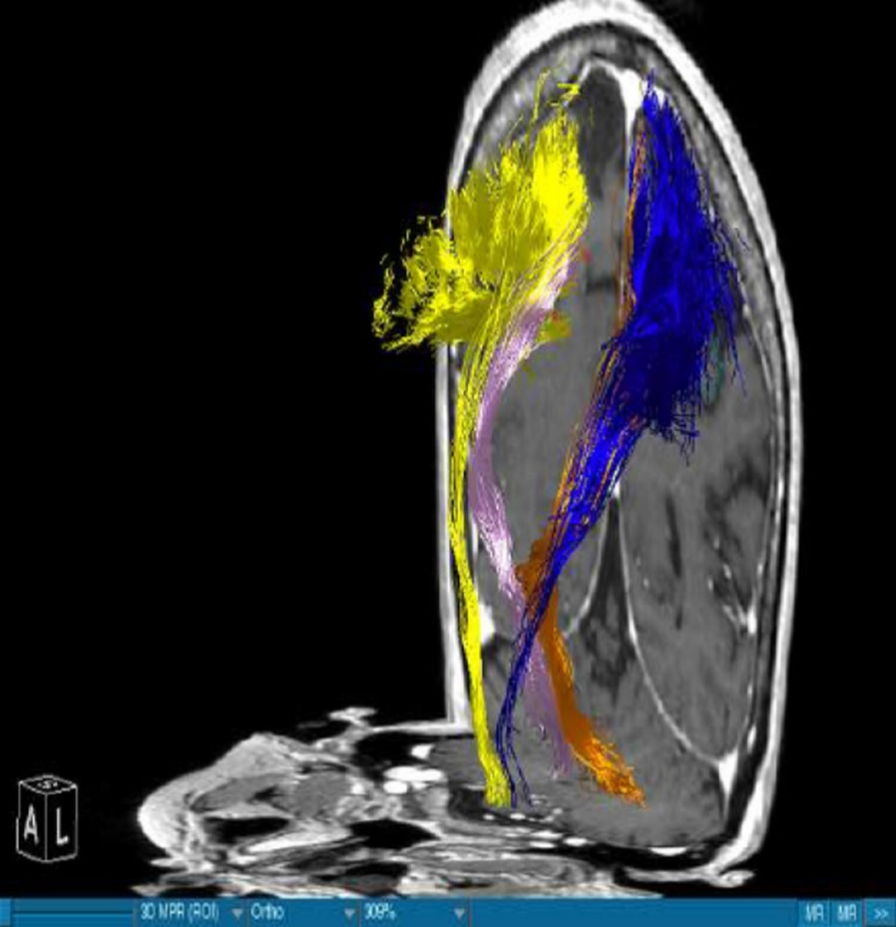
T1-image, semilateral view from *left*, Parkinson’s disease. *Blue* left corticospinal tract, *Yellow* right corticospinal tract, *Gold* left dentatorubrothalamic tract, *Bright purple* right dentatorubrothalamic tract

**Fig. 4:**
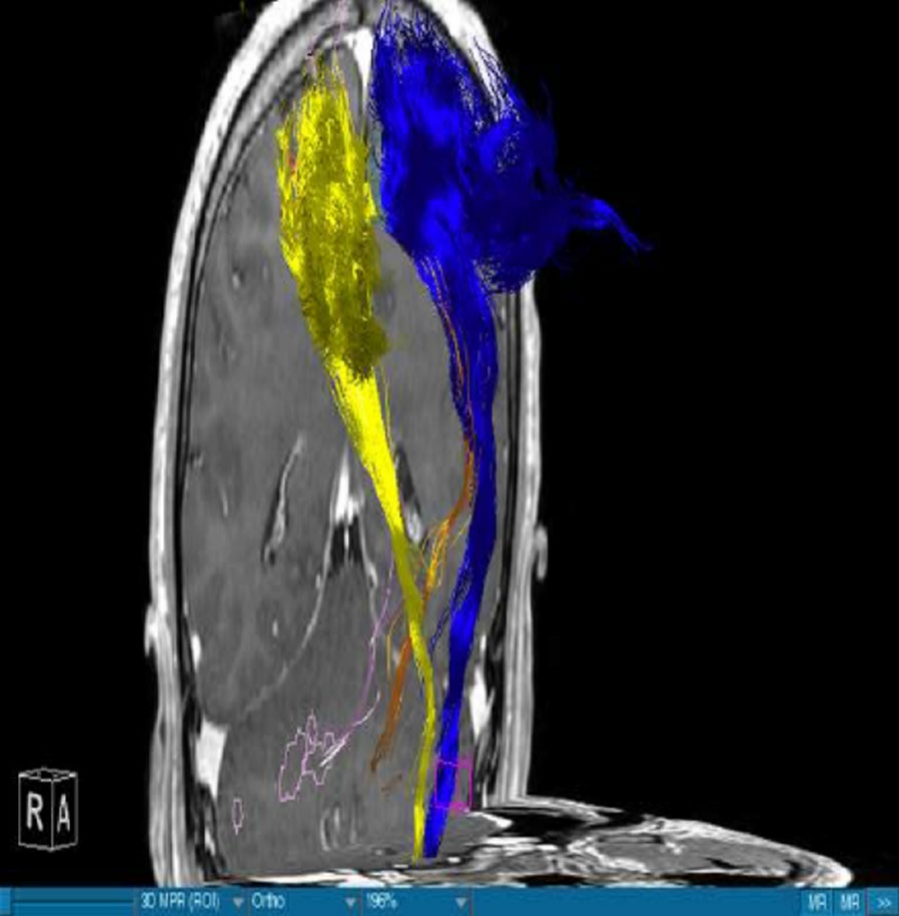
T1-image, semilateral view from *right*, Parkinson’s disease. *Blue* left corticospinal tract, *Yellow* right corticospinal tract, *Gold* left dentatorubrothalamic tract, *Bright purple* right dentatorubrothalamic tract

**Fig. 5:**
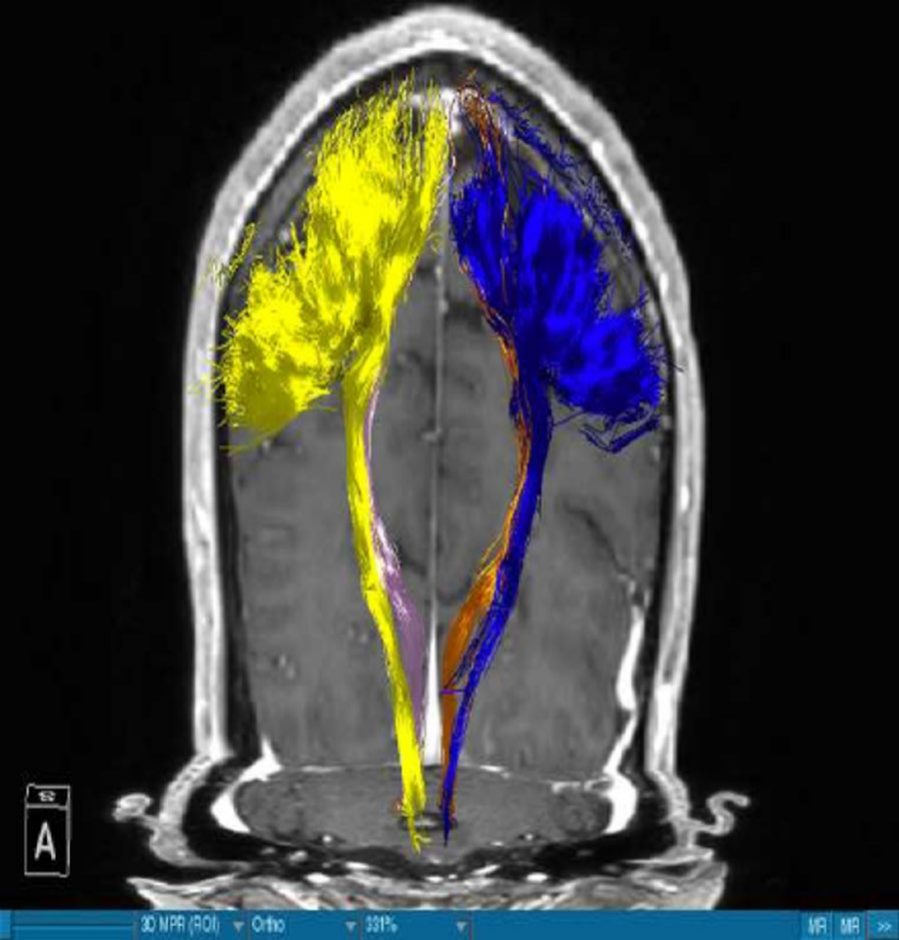
T1-image, anterior view, Parkinson’s disease. *Blue* left corticospinal tract, *Yellow* right corticospinal tract, *Gold* left dentatorubrothalamic tract, *Bright purple* right dentatorubrothalamic tract

Using DTI we were able to successfully portray fibers of two main WMT, the CST and the DRT, in all our patients. We visualized the medial and lateral parts of the CST by using a ROI which covered the whole MC. A segmentation of the MC was performed in order to enhance the accuracy. The fibers ran from this area of the brain through the internal capsule and they could be followed until their entry in the brainstem. Our main interest was, however, the portrayal of the DRT. This WMT begins in the DN of the cerebellum. The fibers arise from the DN dorsolaterally of the fourth ventricle and project through the superior cerebellar peduncle (Figs. 1, 3). They enter into the contralateral red nucleus after leaving the pons behind. The fibers proceed further to the thalamus, in the ventral anterior and ventral lateral nuclei. From this point they proceed their way to the medial part of the MC next to the midline (Figs. 4, 5). This doesn’t mean there is one axon arising from one point and finishing in another one. It is rather a bundle of fibers. The DRT traverses through his course the posterior subthalamic nucleus, zona incerta and the thalamus. It is situated medially of the CST in his course from the cerebellum to the thalamus (Figs. 2, 5). From the thalamus on, it proceeds medially and posteriorly of the CST further to the MC. From our point of view this is a very important finding. Fibertracking from the MC in direction of the brainstem e.g. in case of the CST might include fibers of the DRT too. It would be difficult for physicians to distinguish between these two tracts. We have to follow the DRT from the cerebellum up to the MC in order to be able to differentiate between DRT and CST. The start of the DRT in the cerebellum and the further course of the CST through the brainstem give us the possibility of differentiation between the two. The fibers were tracked from the thalamus to the MC. We segmented the MC before this step. It is possible to use a ROI instead of the segmented area. From our point of view we can delineate the MC better if we segment it. The differentiation between this tract and other WMTs is possible if we follow the fibers in their whole course from the DN in the cerebellum. Other thalamocortical fibers do not have their starting point there. We think that fibers which run through these points belong to the DRT and not to another tract. In our study the results of these WMT were anatomically reproducible. The relation between these two WMT did not differ from one patient to another. We have to say, however, that the density of the fibers was not the same in all our patients. Some of the patients presented with a much higher density of fibers like the patient in Fig. 3 whereas other patients presented thinner WMTs like the patient in Fig. 4.

## Discussion

### DTI-introduction

DTI is a technique which can visualize WMT in vivo in healthy and non healthy patients by measuring anisotropic water diffusion of the brain [18–20]. This technique has been used increasingly in recent years by neurosurgeons to identify WMT and deal with tumours in eloquent areas of the brain in order to reduce postoperative morbidity [21]. Some of these WMT include the visual pathway, the arcuate fasciculus or the CST. Additionally DTI might be a useful tool in patients with PD. It has a good sensitivity and specificity to differentiate between healthy subjects and de novo patients [3, 22].

### DRT-introduction

The portrayal of other WMT like the DRT might be also of considerable importance for neurosurgeons during DBS in patients with PD for tremor control. This tract has its starting point in the cerebellum in the DN, then it continues to the contralateral red nucleus and ventral anterolateral nuclei of thalamus from where the fibers proceed to the MC [23]. This WMT possesses not only motoric but also linguistic and cognitive functions [24]. Injury of this tract might lead to movement disorders like ataxia or tremor [25–27]. It has been suggested that damage in the white matter integrity in the posterior portion might be responsible for motor symptoms while alterations in the ventral part might be related to communication behaviour in patients with autism [23].

### DRT-anatomy and review of the literature

There have been two publications which suggest that tremor control with low voltages in the posterior subthalamic region might be attributed to stimulation of the DRT [11, 28]. As this region is a station of the DRT in his course to the thalamus, this might be very well possible. Coenen et al. [11] have provided a case where stimulation of this tract was directly involved in tremor reduction. Furthermore they emphasize that we are actually dealing with one fiber structure during DBS and this is the DRT [29]. A stimulation might not be sufficient if it doesn’t affect enough fibers of DRT. If it is far anterior tremor reduction might not be satisfactory and if it is far posterior we might stimulate the medial lemniscus [29]. By knowing the exact position of the electrode and its angle to the stimulated cerebral structure we might be able to predict the necessary voltages needed for adequate results. Depending on the stimulated part of DRT we might achieve different results in tremor control and avoid side effects. That means we might be able to increase the voltage more if the electrode is far enough of certain structures like the CST. We have to be aware of the fact that the CST runs laterally of the DRT (Fig. 2).

### DRT-stimulation site

Depending on the position of the electrode the CST might be affected by a high voltage if the electrode is situated in the lateral portion of the DRT or by the electrode itself if this one is localized outside the DRT. There have been suggestions that the optimal position is inside in the anterior third of the DRT [29]. By means of DTI, it might be, however, difficult to portray WMT completely and in that case it might be difficult to determine the anterior third of the DRT exactly. Another study, however, published by another group suggests that tremor reduction is better achieved if we stimulate directly inside the DRT [30]. Another option seems to be the stimulation in the quadrant posterior, inferior and lateral to it [30]. Although the DRT seems to be an important structure in DBS we have to emphasize the fact that the optimal electrode placement is not so clear. However, we deem necessary to emphasize the fact that the DRT proceeds next to the CST after leaving the thalamus (Figs. 1, 2, 3, 4, 5). A stimulation in that area might have eventual unintended side effects in the patients. We propose every stimulation should occur caudally to this cerebral area. Therefore, from our point of view, a depiction of both tracts, the DRT and CST, before DBS is important. If the electric field influences neighbouring regions like the CST, it might compromise our results. Our results show that the CST is localized laterally to the fibers of DRT (Figs. 1, 2, 3, 4, 5). Therefore, we need to be very cautious when we stimulate laterally in order to avoid unwanted side effects. The accurate knowledge of the electrode placement after DBS might give additional information of tremor control in patients with PD or other forms of tremor. Another important issue which we need to mention is that we might not be able to stimulate the DRT everywhere in his course e.g. in the DN or red nucleus and achieve the same results as when we stimulate the subthalamic nucleus. By knowing the exact position of the electrode and its angle to the stimulated cerebral structure we might be able to predict the necessary voltages needed for adequate results. Depending on the stimulated part of DRT we might achieve different results in tremor control and avoid side effects. That means we might be able to increase the voltage more if the electrode is far enough of certain structures like the CST. A recently published study, however, has revealed that stimulation closer to the DRT doesn’t provide better results than more distant stimulation [30]. However, even if the results don’t seem to be better we should be carefully taking into account the volume of tissue activated in order to avoid side effects [31]. Besides to its motoric functions this WMT possesses linguistic and cognitive functions too [24]. An injury of DRT leads to cognitive and behavioural deficits, speech impairment or emotional lability [24]. Patients with PD develop these symptoms in the course of their disease, therefore a degeneration of this tract might explain this phenomenon. Further work needs to be done in order to deal with these symptoms.

### PD affects the thalamus

Postmortem studies show that PD affects the thalamus in its own way [3]. It can provoke a degeneration or disruption of the microstructural integrity of the thalamus [3, 32, 33]. As we mentioned above the thalamus is a region which is crossed by the DRT. A degeneration or disruption of this region might induce a degeneration or disruption of this WMT and ultimately be one of the reasons for PD. It seems not to be known how many fibers of this tract need to be stimulated in order to have satisfactory results [29]. However, this might be dependent on the progress of the disease and the grade of fiber degeneration.

### DTI-limitations

DTI was used for the visualization of DRT and CST. It has, however, some limitations. It is difficult to determine with accuracy the starting point and ending point of the fibers [34, 35]. The resolution of crossing or kissing fibers remains also a problem which should be taken into account [34, 35]. Nevertheless, the portrayal of these tracts was reproducible in nine different patients on different times. The DRT were all similar in their form and their relation to the CST, so we believe that the accuracy of our results remains high.

## Limitations of the study

The authors are aware of the limitations of the current study. On one hand we are portraying here a small number of patients and on the other hand there is no control group. However, we have to say that, when compared to the existing literature, we found that our results concerning the depiction of the DRT are in accordance with the results of other authors who have worked in this direction. Coenen et al. [17] confirm that the DRT traverses the thalamus, zona incerta and the STN. They add that this WMT is located in the proximity of the CST. In our cases Figs. 1, 2, 3, 4 and 5 we localised the DRT medially to the CST. Other studies name the superior cerebellar peduncle, the DN and the thalamus as structures traversed by the DRT [25]. We can confirm that. Despite the small group of patients our results are in accordance with the existing literature.

On the other hand, further limitations concern the portrayal of WMT by means of DTI, e.g. the portrayal of crossing or kissing fibers might be difficult and the fact that the starting and ending point of the WMT cannot be visualized correctly [34, 35]. When two WMT are running in the proximity of each other then DTI will have to distinguish which fibers belong to one tract and which fibers belong to the other. Therefore, there is a risk that fibers of the smaller tract might be included to the fibers of the other one. Making the difference between the two might be difficult for physicians. In our case we have fibers of the DRT and CST. In the Figs. 1, 2, 3, 4 and 5 we see different tracts by using different colours, however, we have to take into account that some fibers might be attributed to the bigger tract due to the limitation mentioned above. Patient motion might be another limitation of the DTI [35]. Diffusion MRI is very sensitive to motion [35]. Therefore, DTI might be difficult in patients suffering from PD.

## Conclusion

Our study shows that portrayal of DRT fibers is possible for everyday routine use with a little amount of time. All the results are easily reproducible. From our point of view it is necessary to visualize the CST along with the DRT before surgery in order to have a better idea about the relation between these two WMT which are situated very close to each other in order to avoid unwanted side effects postoperatively (Fig. 5). Identification of DRT can be useful for physicians in controlling tremor.

## Abbreviations

ADC: apparent diffusion coefficient
ASSET: array spatial sensitivity encoding technique
CST: corticospinal tract
DBS: deep brain stimulation
DN: dentate nucleus
DRT: dentatorubrothalamic tract
DTI: diffusion tensor imaging
EPI: echo planar imaging
FA: fractional anisotropy
FACT: fiber assignment by continuous tracking
FOV: field of view
MC: motor cortex
MRI: magnetic resonance imaging
MS: multiple sclerosis
PD: Parkinson’s disease
ROI: region of interest
WMT: white matter tracts
